# Smoker's Palate: An Often Misunderstood Benign Lesion of the Oral Cavity

**DOI:** 10.7759/cureus.48868

**Published:** 2023-11-15

**Authors:** Hussain Ali S John, Rishika Dakhale, Shweta Sedani, Kajal P Ahuja

**Affiliations:** 1 Oral and Maxillofacial Surgery, Sharad Pawar Dental College and Hospital, Datta Meghe Institute of Higher Education and Research, Wardha, IND; 2 Public Health Dentistry, Sharad Pawar Dental College and Hospital, Datta Meghe Institute of Higher Education and Research, Wardha, IND; 3 Conservative Dentistry and Endodontics, Sharad Pawar Dental College and Hospital, Datta Meghe Institute of Higher Education and Research, Wardha, IND; 4 Orthodontics and Dentofacial Orthopaedics, Sharad Pawar Dental College and Hospital, Datta Meghe Institute of Higher Education and Research, Wardha, IND

**Keywords:** nicotina stomatitis, smoker, smoking, nicotina palatini, smoker's palate

## Abstract

Smoker's palate is a type of lesion that occurs on the mucosa of the hard and soft palate almost exclusively in smokers. This lesion is prevalent in smokers who practice reverse smoking and less common in cigar and cigarette smokers. The lesion known as smoker's palate is also referred to as nicotina stomatitis and nicotina palatini, which suggests the role of nicotine in the manifestation of the lesion, but this is a misnomer as the lesion occurs due to the impact of heat coupled with the irritation caused by agents such as tobacco and marijuana; there is no role of nicotine in it. Patients who notice this lesion tend to misinterpret it as an early manifestation of squamous cell carcinoma. Although this is not true, this can be an excellent opportunity to counsel patients regarding the ill effects of smoking and guide them to quit the habit, citing that it causes cancer. This article presents a case report of a 27-year-old male with a smoker's palate. This article also highlights the importance of dental practitioners in diagnosing the lesion and how patients often misunderstand this lesion as a severe condition.

## Introduction

Smoker's palate, which is also known as nicotina stomatitis, nicotina palatini, smoker's keratosis, etc., is a lesion that is commonly seen in smokers [1]. It is often an asymptomatic and benign lesion that involves the hard palate and the anterior part of the soft palate; the anterior part of the hard palate is rarely affected [1,2]. The palate becomes pale due to keratosis, and discrete reddish-colored papules and petechiae may be seen; an erythematous irritation followed by a whitish palatal mucosa may be seen, and the rest of the oral mucosa may show dark brown pigmentation. The classification based on the progression of smoker's palate was given by Greenburg et al., who classified the lesions as mild, moderate, and severe. The lesions are classified as mild when red, dot-like structures are seen on a pale, blanched palatal surface, moderate when there is a characteristic elevation along with umbilicals in the center, and severe when several papules of more than 5 mm or umbilicals of more than 2-3 mm are seen [3-5]. The occurrence of the lesion is a result of the application of heat over a period of time; inflammation of the minor salivary glands present in the hard and soft palate occurs. The anterior part of the hard palate is not affected as not many minor salivary glands are present in that location. Tobacco and marijuana also act as an irritant and act synergistically with heat, which also contributes to the presentation of the lesion [1-3,6]. This lesion is often an accidental finding during a dental examination [1,4]. No investigations are necessary unless carcinoma is suspected. The potentially malignant lesions must be ruled out by taking an accurate case history and performing a thorough clinical examination. Smoker's palate can be misdiagnosed as erythroplakia; speckled white plaques on the palatal mucosa are commonly seen in erythroplakia, but not in smoker's palate. The lesion is benign and does not require any treatment, and it heals spontaneously after abstinence from smoking for about a few weeks [1-3].

## Case presentation

**Figure 1:**
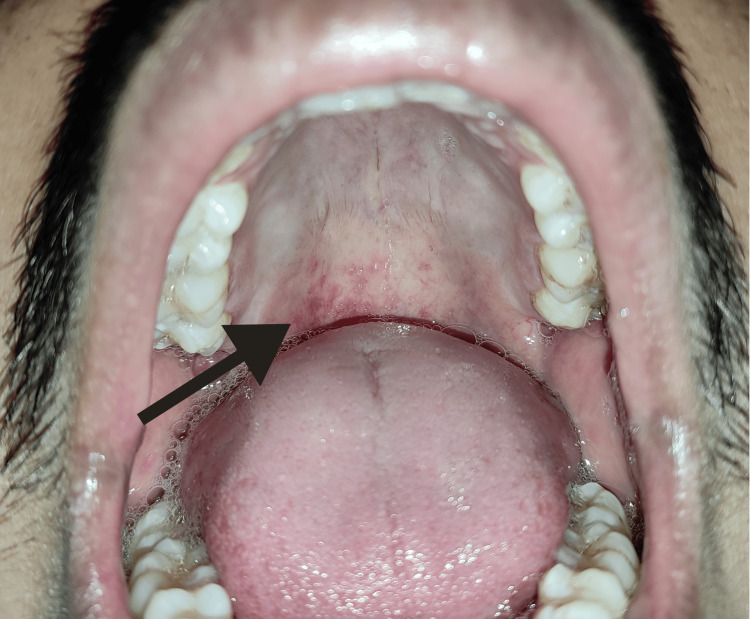
Intra-oral examination of the patient showing inflamed minor salivary glands on the posterior region of the hard palate (arrow) Informed consent of the patient was taken before taking the photo of the lesion.

A 27-year-old male reported to the oral medicine outpatient department in a tertiary care center in Wardha, India, with the chief complaint of reddish spots on the back of his throat. The patient first noticed the small, reddish-colored spots on his palate 15 days ago while brushing his teeth. The patient was not on any medication, and dental history was insignificant. There was no pain or burning sensation associated with the lesion. On intra-oral examination, multiple discrete, elevated, red, inflamed orifices of minor salivary glands of about 3-4 mm in diameter were seen on the posterior one-third region of the hard palate and the junction of the hard and soft palate. The palate was pale in color, and no tenderness was present. The patient had a history of smoking five cigarettes a day for seven years and consuming alcohol once a week for five years. Figure 1 shows the intra-oral examination of the patient.

Based on the clinical examination and habit history given by the patient, a diagnosis of a grade II smoker's palate was made. The patient was made aware of the etiology of the lesion, and no investigations were advised. The patient was advised to stop the habit and was ensured that the lesion would heal spontaneously within 2-4 weeks of not smoking. He was subsequently referred to the tobacco cessation center of the hospital for further counseling. The patient was previously highly anxious as he had assumed that he had contracted squamous cell carcinoma because of his habit. Still, when he was made aware by the practitioner regarding the nature and mechanism by which this lesion occurs, he was relieved and motivated to quit the habit of smoking.

## Discussion

Smoker's palate is considered one of the most common oral diseases caused by smoking [1,3,7]. Although the word "nicotine" is often attached whenever this lesion is mentioned, nicotine has nothing to do with it; the term nicotina stomatitis was coined by Kurt Thoma as patients who displayed shared symptoms of this lesion had a history of tobacco usage [4]. The presentation of the lesion is caused by heat and irritants such as tobacco and marijuana [1,3]. This lesion was first mentioned in the literature in 1926 [8]. Schwartz [2], in 1965, described a few of such lesions as precancerous in nature and believed them to be due to chemical injury to the glandular part of the palatal mucosa. Reverse smoking is a common practice in several endemic areas around the world [9]. In India, the use of hand-rolled homemade cigars is more prevalent. Although this lesion is more common in males, certain studies have found that in certain endemic regions, it is common in females, contrary to popular belief. It is a well-established and socially acceptable habit among adult females in such areas [9,10]. Because the lesion is associated mainly with heat, it can occur in occasional and non-smokers as well. A 75-year-old female reported for a routine dental checkup and had mucosal changes similar to those seen in smoker's palate; the cause of the lesion was her consumption of a lot of hot beverages [11]. The lesion can be mistaken for squamous cell carcinoma by the patient. Although the lesion can be easily diagnosed with the help of the patient's history and a thorough clinical examination, a biopsy should be performed in case the lesion is a result of reverse smoking or if it is a symptomatic lesion [12,13]. The histopathological examination of this lesion usually shows hyperkeratotic changes, epithelial dysplasia, intraductal squamous metaplasia, plasma cell infiltration, and the presence of mucous cysts. The differential diagnosis includes leukoplakia, erythroplakia, candidiasis, and oral squamous cell carcinoma [8]. The definitive treatment of choice for smoker's palate is the cessation of the habit, which results in the lesion healing on its own within a few weeks of quitting [1-3]. These lesions are often accidentally discovered during a routine dental examination; it is the professional duty of the clinician to perform subsequent investigations to identify and manage such lesions [2].

## Conclusions

Smoker's palate is a benign and often asymptomatic lesion of the oral cavity that occurs primarily in smokers due to the heat generated by smoking, resulting in hyperplasia, keratinization, and inflammation of the palate. It is often mislabeled as a lesion caused by nicotine. Tobacco and marijuana are irritants that act synergistically with heat, resulting in this lesion. An unaware patient can also mistake this condition for a lesion associated with carcinoma. The lesion heals spontaneously within weeks of quitting smoking.
